# Reply to ‘Comment on ‘The impact of vitamin D pathway genetic variation and circulating 25-hydroxyvitamin D on cancer outcome: systematic review and meta-analysis’’

**DOI:** 10.1038/bjc.2017.185

**Published:** 2017-06-22

**Authors:** L Zgaga, P G Vaughan-Shaw, F O’Sullivan, S M Farrington, E Theodoratou, H Campbell, M G Dunlop

**Affiliations:** 1 Department of Public Health and Primary Care, Trinity College Dublin, Dublin 24, Ireland; 2 MRC Human Genetics Unit, Institute of Genetics and Molecular Medicine, University of Edinburgh, Edinburgh EH42XU, UK; 3 Centre for Population Health Sciences, University of Edinburgh, Edinburgh EH164UX, UK

**Sir**,

We thank Dr Braillon for his interest in our recent systematic review and meta-analysis, which examines published evidence of the relationship between cancer survival outcome, vitamin D concentration and genetic variation in vitamin D-related pathways. Braillon (2017) raises the issue of the cut-offs in plasma 25-hydroxyvitamin D (25(OH)D3) concentration that we use as vitamin D ‘categories’. These are a consequence of the conduct of original research included in our synthesis; unfortunately, limitations of included studies are an inherent limitation of any systematic review. One particular difficulty when synthesising evidence from vitamin D studies arises from the inconsistent 25(OH)D cut-offs used by researchers in creating statistically favourable conditions for their respective study. Our work explicitly itemises the cut-offs used (Figure 2 in Vaughan-Shaw *et al*, 2017), in contrast to most previous reviews on vitamin D that largely ignore the heterogeneity of category definitions. This leads to between-study heterogeneity and makes the calculation of a meaningful summary estimate challenging. It has been shown that the benefit of vitamin D varies, and appears highest for the most deficient (Talwar *et al*, 2007). Consequentially, heterogeneity affects the ability to assess the true biological significance of vitamin D: different effect sizes are often reported when comparing deficient (e.g., 10 nmol l^−1^) with insufficient (e.g., 35 nmol l^−1^), *vs* comparing sufficient (e.g., 75 nmol l^−1^) with super-sufficient (e.g., 100 nmol l^−1^). It is worth noting that a significant association between 25(OH)D and cancer outcome was found in our review, despite this cut-off heterogeneity.

Actually, one of the key messages of our paper was to highlight the radically different vitamin D category definitions used in the extensive discussion and supplementary materials. Our paper emphasises the need to harmonise category definition in future studies. While using clinical cut-offs of vitamin D deficiency/insufficiency (e.g., per Ross *et al*, 2011) seems to be an obvious solution, two issues undermine this: first, agreement on what these are has not been reached (and may indeed differ for different outcomes), and second, due to large population differences in 25(OH)D concentration the distribution of participants to any preset categories may not be balanced, thereby negatively impacting on the statistical power. Whether distributing subjects into defined 25(OH)D categories is appropriate in any analysis is another issue. We found that most studies (90%) used categories defined by intervals of plasma vitamin D. However, we maintain that incorporating 25(OH)D level as a continuous variable in any analysis is vastly preferable, because it can account for non-linear vitamin D effects.

Dr Braillon further states that we did not adjust for main confounding clinical variables. Indeed, some of the early studies included in our review failed to adjust for some confounders. To mitigate against this, we used the most fully adjusted estimates in the meta-analyses and systematically assessed study quality – confounding factor assessment being one of our quality criteria. Finally, we performed a stratified analysis based on original study quality, thereby using all reasonable means to address this issue in synthesis. Nonetheless, we agree that confounding remains an issue for any observational study (or review), as many cancer risk factors are also associated with vitamin D deficiency. In these circumstances, demonstrated association with genetic factors involved in vitamin D metabolism offers an important new perspective that supports a causal link for vitamin D deficiency and cancer outcomes, in absence of RCTs. This is why we conclude that our findings provide powerful background rationale to instigate clinical trials to prospectively investigate the effect of vitamin D on cancer outcomes.

Finally, in our view personalised medicine converges on the joint consideration of phenotypic and genotypic data, since these are biologically inseparable. Outcomes are affected by genotypes, phenotypes and also by their complex interaction. In our review, we reported on the impact of vitamin D phenotype (25(OH)D concentration) and vitamin D-related genotype on cancer outcome. Previously, we reported an interaction between genetic variation at the vitamin D receptor gene and vitamin D levels which influenced colorectal cancer survival (Zgaga *et al*, 2014). Together, these support the premise that vitamin D supplementation trials should be stratified by genotype. Truly personalised medicine will enable modification of the phenotype according to the genetic predisposition, via more accurate subgroup classification.
